# Randomized communication nudges to increase primary care engagement among Medicaid enrollees in Maine

**DOI:** 10.1093/haschl/qxag100

**Published:** 2026-06-24

**Authors:** Sarah H Gordon, Kevin H Nguyen, Breanne Biondi-Bonetti, Jacob Wallace, Benjamin D Sommers, Anna Goldman

**Affiliations:** Department of Health Law, Policy, and Management, Boston University School of Public Health, 715 Albany Street, Boston, MA 02118, United States; Department of Health Law, Policy, and Management, Boston University School of Public Health, 715 Albany Street, Boston, MA 02118, United States; Crown Family School of Social Work, Policy, and Practice, The University of Chicago, Chicago, IL 60637, United States; Department of Health Policy and Management, Yale School of Public Health, New Haven, CT 06510, United States; Department of Health Policy and Management, Harvard T. H. Chan School of Public Health/Department of Medicine, Brigham & Women's Hospital, Boston, MA 02115, United States; Division of General Internal Medicine, Boston University School of Medicine/Boston Medical Center, Boston, MA 02118, United States

**Keywords:** primary care, RCT, medicaid, letters, randomized intervention

## Abstract

Despite the importance of primary care in disease prevention, few studies have examined behavioral interventions to increase primary care engagement. In partnership with the Maine Department of Health and Human Services, we implemented a randomized mail-based outreach strategy to connect newly enrolled adults in MaineCare Primary Care Case Management with primary care providers. From March 16, 2021, through January 1, 2022, members were randomized to receive either (1) a standard text-heavy outreach packet (control arm) or (2) the standard packet plus 2 visually simplified mailers that emphasized the value and low cost of a primary care check-up and listed a single primary care provider near the enrollee's home (intervention arm). We found that over 12 months of follow-up, the intervention had no significant effects on the rate or number of primary care visits, emergency department visits, or inpatient hospitalizations. We also observed no significant impact on Medicaid enrollment or recommended primary care services, including receipt of flu shots, cholesterol screenings, or mammograms. Results suggest that barriers targeted by the mailers were not primary drivers of primary care engagement, and other strategies are needed to help connect new Medicaid enrollees with primary care.

## Introduction

In January of 2019, Maine expanded its Medicaid program, MaineCare, to low-income adults with family incomes below 138% FPL, after a ballot initiative was approved in November of 2017.^1,2^ A central goal of the state's Medicaid expansion was to connect newly enrolled individuals with primary care services. Primary care can improve health outcomes through increased use of preventive services, such as cancer screenings and vaccination, as well as chronic disease management for conditions such as diabetes, asthma, and heart disease.^3,4^ Although evidence is mixed and limited, several studies suggest that appropriate primary care use may divert patients from high-cost emergency department use, resulting in system-wide cost savings.^5,6^ While health insurance expansions have been shown to increase utilization of preventive care and chronic disease management, 1 multistate study previously found that only 62.2% of adult Medicaid enrollees had at least 1 primary care visit in 2019, leading to missed opportunities to improve population health.^7-9^

Under Primary Care Case Management (PCCM) models, state Medicaid agencies contract directly with primary care providers to manage the health of a panel of patients, combining fee-for-service provider payments with additional per member per month fees to cover the cost of proactive management of patient care.^10^ At the time of expansion, the vast majority of MaineCare enrollees were in PCCM, as enrollment was mandatory for low-income adults, children, and those with disabilities. Individuals newly enrolled through Medicaid expansion were enrolled in PCCM. In Maine, new PCCM enrollees were requested to select a primary care provider (PCP) from a lengthy packet with a provider directory; however, MaineCare staff reported that less than half of PCCM members contacted MaineCare to select a PCP.

This low PCP selection rate may have been explained by barriers that are well-described in the behavioral economics literature: The number of provider options may have induced choice overload, in which an abundance of options resulted in no choice at all.^11^ Hassle costs, such as requiring enrollees to place phone calls or wait on hold, may have significantly decreased enrollees’ motivation to choose a PCP.^12,13^ These effects may have been magnified in a population with low levels of health literacy and limited experience with health insurance.^14,15^ While mail-based outreach is a well-established communication modality with demonstrated effectiveness in Medicaid populations, participants in an advocacy roundtable noted that welcome packages sent by mail to new Medicaid enrollees were often too long and complex, suggesting that providing visually oriented, accessible, and simple communications with member-specific information may be more effective forms of written outreach.^16-18^ Prior studies have found that mail-based behavioral “nudges” increased engagement with health insurance.^19-23^ However, none of these studies targeted the multi-step process of contacting, scheduling, and attending an appointment with a primary care provider.

From March 16, 2021, to December 17, 2021, we conducted a randomized controlled trial in partnership with the Maine Department of Health and Human Services, in which we tested a communication outreach strategy to connect new MaineCare enrollees with primary care. Enrollees in MaineCare's PCCM program either received the standard, lengthy packet with small dense black and white text that all new Medicaid enrollees receive (control arm), or the standard mailer *plus* 2 additional visually simplified and personalized mailers that emphasized the value and low cost of a check-up and listed a single default primary care provider within 30 miles of the enrollee's home (intervention arm).

The intervention drew on 4 behavioral mechanisms identified in the nudge literature: (1) default assignment, which leverages inertia by making PCP enrollment automatic absent active opt-out; (2) choice simplification, which reduces decision paralysis from provider choice overload; (3) friction reduction, which lowers the hassle costs of PCP selection; and (4) salience enhancement, which improves attention to actionable information about the value of primary care through visual simplification and personalization.^13,24-26^ Collectively, we hypothesized that these mailers would increase primary care use due to the personalized messaging, simplified language, and reduction in provider options; a decrease in the complexity of the PCP selection process; the salience of a conveniently located provider's name and contact information; inertia from the “opt-out” framework; and the information on primary care's value.^27,28^ We further hypothesized that the simplified mailer may in turn reduce inpatient and emergency department use for conditions potentially addressable in primary care settings, and that receipt of recommended preventive services would increase among those that received the intervention.

## Methods

### Study design

The experimental design was an individual-level randomization among newly enrolled members of the MaineCare Primary Care Case Management Program (PCCM). To be included in the study sample, individuals were required to be between the ages of 19 and 64 and not have been enrolled in PCCM within the last year. Our intervention was implemented from March 16, 2021, through January 1, 2022. Each enrollee was then followed for 2 years from the date of their last mailer (through January 1, 2024, at the latest).

Upon enrollment in MaineCare, new PCCM members received standard enrollment paperwork, including a lengthy enrollment packet with dense black and white type. This enrollment packet required new members to select a PCP from an enclosed directory and inform MaineCare of their choice by phone or by mail. The provider directory listed providers who were not guaranteed to be accepting new Medicaid patients. It included a provider choice form on the second page of the packet on which the enrollee could write in 3 provider names from the directory.

Those in the control group of our study received only the standard enrollment packet. Those in the intervention group also received the standard enrollment packet but then received 2 additional mailings from MaineCare. These mailers were printed on thick cardstock with brighter colors, larger font sizes, and more blank space than the standard packet. Approximately 7 days after the standard enrollment packet was sent, members were sent a simplified, personalized, visually appealing mail card that listed a PCP within 30 miles of the enrollee's home. If the enrollee did nothing, the listed provider was automatically assigned to them as their PCP. If they wanted to change their PCP, they could contact MaineCare to do so. This approach retained an enrollee's choice of provider but applied an “opt-out” framework to PCP selection. Assigned PCPs were selected by MaineCare staff from a list of MaineCare-enrolled providers accepting new patients.

The mailer read: “[Name] is enrolled in MaineCare with a program that helps manage your health care needs. Developing a relationship with a Primary Care Provider (PCP) is a step toward better health.” The intervention mailer also emphasized the low cost of seeing their doctor: “There should be no cost, unless you have a copay;” and included a section titled “How can my PCP help me?” Enrollees were then advised to call their PCP to make an appointment. The mailer included guidance for how to opt out of the assigned PCP if desired: “If this isn’t your PCP or you would like to choose a different PCP, do one of the following…” The mail card then listed several ways for the enrollee to contact MaineCare, including an enclosed prepaid mailer that enrollees could send back to inform MaineCare of their preferred PCP.

Approximately 14 days after the new enrollment packet was sent, enrollees in the intervention group were sent a second reminder mail card. This mail card read “[Name] has health insurance with MaineCare. Connecting with a primary care provider (PCP) is the next step to keeping you healthy! It's time to call your PCP about an appointment.” The mail card also noted that the enrollee may be able to see their PCP from the comfort of their home via telehealth and provided instructions for how to opt out of their listed PCP and a prepaid mail card to do so. If enrollees did not opt out, they were assigned the listed PCP. Example mailers and further details on the randomization protocol are provided in Appendices 1 and 2.(To access the Appendix, click on the Details tab of the article online.)

### Data and sample

Our study sample included new enrollees ages 19-64 in the MaineCare PCCM program. PCCM enrollment in Maine was mandatory for low-income adults, children, and those with disabilities during our study period. The study sample included all eligibility pathways and was not limited to those who enrolled through the state's Medicaid expansion. Those who had been in Medicaid within the past year were excluded from the study, as were those who had received new enrollee packets more than 1 day before randomization.

The Office of MaineCare Services provided a dataset on study participants that included the enrollee's Medicaid ID, treatment assignment (intervention vs control), assigned provider and practice, dates mailers were sent, dates enrollees responded to the mailer, and how they did so (mail vs phone). After the intervention ended, enrollees were followed for 12 months from the date of their last mailer using Maine Medicaid administrative claims data from 2019 to 2023 that were linked to the randomized sample using the Medicaid beneficiary ID.

### Measures

The primary outcome of interest was the rate of primary care visits within 365 days of the last mailer sent. Primary care visits were identified following a method developed by the Maine Quality Forum for state-mandated reporting on primary care spending using Maine Medicaid claims data.^29^ In brief, primary care visits were defined as a visit to a provider with a primary care taxonomy code where at least 1 qualifying primary care service was rendered (ie, immunizations, evaluation and management services for office visits, preventive visits) based on relevant procedural terminology (HCPCS) or procedure codes (CPT). Federally qualified health centers and rural health centers were classified as primary care providers following Maine Quality Forum's approach.

Other secondary outcomes included whether enrollees contacted MaineCare in response to the mailers (ie, via phone, email, or mail), the number and rate of emergency department visits, all-cause inpatient hospitalization, and ambulatory care–sensitive admissions. Primary care and emergency department visit rates were assessed 90 and 365 days after the last mailer was sent, while inpatient outcomes were assessed 365 days after the last mailer. We also examined receipt of an annual flu shot, annual cholesterol screening, any mammogram for females ages 50-64, and any hemoglobin A1C and microalbumin testing for patients with a diagnosis of type 1 or type 2 diabetes, and probability of Medicaid coverage loss, all within 365 days of enrollment. Appendix 3 contains additional details on outcome definitions. Covariates included gender, age, race, ethnicity, and ZIP code-level rurality measured by Rural-Urban Commuting Area Codes.

### Statistical analyses

Our trial and analysis plan was pre-registered on the American Economic Association Randomized Controlled Trials Registry on December 6, 2023. We first compared characteristics of our intervention and control groups using 2-sided *t*-tests and Pearson's chi-square tests to assess whether enrollees were balanced across observable characteristics. We then compared primary and secondary outcomes in the intervention vs control groups using Pearson chi-square tests for binary outcomes and *t*-tests for continuous outcomes.

To improve the precision of our estimates and address any residual imbalance in observable features, we also estimated adjusted logistic regression models to compare outcomes between groups, controlling for gender, age, race, and ethnicity. Odds ratios were converted to marginal effects for interpretability and standard errors were clustered at the ZIP code level. In supplementary analyses, we tested whether the effect of the intervention varied significantly by rurality of an enrollee's ZIP code.

### Limitations

This study has several limitations. First, we were unable to assess baseline health status from claims data prior to enrollment because by design, all enrollees in our sample were new to the MaineCare PCCM program. Second, due to eligibility system limitations, the intervention group also received the standard mailer packet that instructed them to select a MaineCare PCP in addition to the mailers, which may have been confusing to some enrollees leading to increased hassle costs and potentially biasing our findings toward the null. Third, those who responded to the first packet or mailer did not receive the remainder of the mailers, meaning that not everyone in the intervention group received all 3 mailers. Lastly, we were not able to pilot the redesigned mailers to obtain member input on accessibility, and they were only provided in English.

## Results

Our final sample consisted of 859 enrollees in the intervention group and 3342 enrollees in the control group. Thirteen enrollees were excluded because they were deemed ineligible after initial PCCM enrollment (Appendix 4). Enrollees in the intervention and control groups were not statistically different from each other with respect to age, gender, race, or ethnicity (Table 1).

**Table 1.** Demographic characteristics of intervention and control primary care case management enrollees in Maine.

	Intervention (*n* = 858)	Control (*n* = 3329)	*P*-value
Age, mean (SD)	38.5 (12.9)	38.9 (13.1)	.399
Gender, *n* (%)			.293
Male	425 (49.5)	1582 (47.5)	
Female	433 (50.5)	1747 (52.5)	
Race, *n* (%)			.707
White	638 (74.4)	2462 (74.0)	
African American	33 (3.8)	133 (4.0)	
Other race	20 (2.3)	102 (3.1)	
Unknown race	167 (19.4)	632 (19.0)	
Ethnicity, *n*(%)			.811
Non-Hispanic	766 (89.3)	2946 (88.5)	
Hispanic ethnicity/unknown	92 (10.7)	383 (11.5)	

Source: Analysis of MaineCare administrative Medicaid claims from March 16, 2021, to January 1, 2024. *P*-values based on independent samples *t*-test for age (continuous) and Pearson's chi-square for all other categorical variables. American Indian/Alaska Native, other race, and unknown race were collapsed into a single group as were Hispanic and unknown ethnicities due to sample sizes <10 and cell suppression policies.

Enrollees in the intervention group, who had a PCP assigned to them, were not required to contact MaineCare unless they wanted to change or confirm their PCP. In this group, 23.7% contacted MaineCare for any reason, compared to a 41.1% contact rate among enrollees in the control group, who were only instructed to contact MaineCare to select a PCP (*P* < .001). Among those who contacted MaineCare in the intervention group, 59.8% did so via phone, 13.0% via mail and phone, and 27.9% via mail only. In the control group, 71.4% contacted MaineCare via phone and 28.6% via mail.

**Figure 1.**
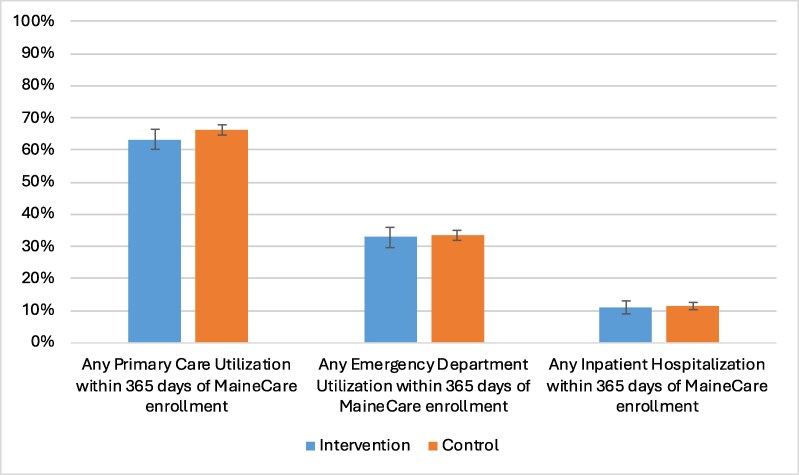
Rates of health care use among intervention and control among Primary Care Case Management (PCCM) members in Maine **Source:** Analysis of MaineCare administrative Medicaid claims from March 16, 2021, to January 1, 2024. **Notes**: Outcomes were assessed within 365 days of the date the last mailer was sent. Differences in the utilization measures shown in Figure 1 were not statistically significant between intervention and control groups at a *P*-value level of .05.

We found that 63.2% of enrollees in the intervention group and 66.2% of enrollees in the control group had at least 1 annual primary care visit within 365 days of the last mailer sent date; these rates were not statistically different from each other (Difference −3.0, *P* = .092) (Figure 1, Appendix 5). Annual rates of any emergency department use were 32.9% in the intervention group and 33.6% in the control group, which was not a statistically significant difference (*P* = .679). Rates of annual inpatient admissions were 10.8% in the intervention group and 11.5% in the control group, this difference was also not statistically significant (*P* = .584) (Figure 1).

Table 2 shows the unadjusted mean values and effects of the intervention from adjusted models. We observed no significant differences across intervention and control groups in the adjusted rates of primary care use, emergency department visits, and inpatient hospitalizations within 365 days of when the last mailer was sent (Figure 1). We also observed no significant effects of the intervention on rates of Medicaid coverage loss or ambulatory-care sensitive admissions in unadjusted comparisons or adjusted models (Table 2).

**Table 2.** Rates of MaineCare enrollment and health care use among intervention and control Primary Care Case Management (PCCM) enrollees in Maine.

	Intervention	Control	Unadjusted difference	Adjusted difference (95% CI)	*P*-value
Any MaineCare contact	23.7% (203)	41.1% (1369)	−17.4	−7.2 (−20.4, −13.9)	<.001
Any coverage loss, % (*n*)^a^	5.5% (47)	5.2% (173)	0.2	0.3 (−1.4, 2.0)	.855
Primary Care utilization, % (*n*)					
Visit within 90 days of last mailer sent	39.9% (342)	41.7% (1387)	−1.8	−1.5 (−4.7, 1.8)	.378
Visit within 365 days of last mailer sent	63.2% (542)	66.2% (2205)	−3.0	−2.8 (−6.5, 0.9)	.139
Emergency department utilization, % (*n*)					
Visit within 90 days of last mailer sent	13.1% (112)	13.8% (461)	−0.7	−0.8(−3.5, 1.9)	.552
Visit within 365 days of last mailer sent	32.9% (282)	33.6% (1119)	−0.7	−0.8(−3.8, 2.2)	.601
All-cause inpatient hospitalization within 365 days of last mailer sent, % (*n*)	10.8% (93)	11.5% (383)	−0.7	−0.6 (−3.0, 1.7)	.590
Ambulatory care–sensitive admission within 365 days of last mailer sent, % (*n*)	2.6% (22)	2.8% (93)	−0.2	−0.1 (−1.5, 1.2)	.832

Source: Analysis of MaineCare administrative Medicaid claims from March 16, 2021, to January 1, 2024. Unadjusted differences compared intervention and control groups using Pearson's chi-square tests for binary outcomes and *t*-tests for continuous outcomes. Any coverage loss defined as less than 12 months of MaineCare coverage from the last mailer sent date. The *P*-value for emergency department visits within 365 days was adjusted to account for multiple contrasts. Adjusted differences used logistic models for binary outcomes and negative binomial models for count variables and adjusted for age, sex, race, and ethnicity. Standard errors were clustered at ZIP code level.

^a^ *P* < .05.

Finally, we also observed no significant differences in receipt of recommended preventive care, including flu shots, cholesterol screening, and mammograms across intervention and control groups (Table 3). Among patients with a diagnosis of diabetes, the intervention did not result in any statistically significant changes in receipt of A1C or microalbumin tests (Table 3). In supplementary analyses, we found no differences in the effect of the intervention by rurality (Appendix 6).

**Table 3.** Rates of recommended preventive care services among intervention and control Primary Care Case Management (PCCM) enrollees in Maine.

	Intervention	Control	Unadjusted difference	Adjusted difference (95% CI)	*P*-value
Flu shot within 365 days of (*n* = 4187)	10.4% (89)	11.6% (385)	−1.2	−1.1 (−3.7, 1.6)	.434
Annual cholesterol screening (*n* = 4187)	23.2% (199)	23.5% (781)	−0.3	0.1 (−2.5, 2.7)	.959
Mammogram (*n* = 457)	31.6% (31)	34.3% (123)	−2.7	−2.2 (−12.6, 8.2)	.676
Chlamydia testing (*n* = 445)	12.1% (11)	21.8% (77)	−9.7	−7.3 (−15.9, 1.4)	.099
Hemoglobin A1C for enrollees with diabetes (*n* = 309)	75.4% (46)	74.2% (184)	1.2	0.8 (−9.7,11.4)	.876
Microalbumin for enrollees with diabetes (*n* = 316)	30.3% (20)	40.4% (101)	−10.1	−10.4 (−22.6, 1.8)	.095

Source: Analysis of MaineCare administrative Medicaid claims from March 16, 2021, to January 1, 2024. Unadjusted differences compared intervention and control groups using Pearson's chi-square tests for binary outcomes and *t*-tests for continuous outcomes. Adjusted differences used logistic models for binary outcomes and negative binomial models for count variables and adjusted for age, sex, race, and ethnicity. Standard errors were clustered at ZIP code level.

## Discussion

In this randomized controlled trial in partnership with the Office of MaineCare Services, we found that a mail-based intervention that listed a default PCP for new PCCM enrollees did not increase primary care use or reduce emergency department or inpatient admissions. We also observed no significant effects of the intervention on Medicaid coverage loss or receipt of preventive care. However, enrollees in the intervention arm contacted MaineCare at lower rates, likely because a PCP was automatically listed for them and they were instructed to contact MaineCare only if they wanted to change PCPs. This suggests that the “opt out” framework of PCP assignment increased the proportion of PCCM enrollees with an assigned PCP and may have reduced the administrative burden of selecting a PCP, though did not result in meaningful changes in health care use.

Our findings may be explained by several possible mechanisms. First, the intervention and control groups both received the initial standard mailer, which may have biased our findings toward the null. Second, mail-based interventions may not be effective among enrollees whose mail goes undelivered or unopened. Third, PCP assignment within close geographic proximity may not alter primary care use for patients who already have a usual source of primary care and established care patterns. For enrollees without a usual source of care, the intervention may not have been strong enough to overcome the “shadow prices” of seeing a PCP, such as concerns about cost, scheduling or transportation constraints, low perceived need for care, attitudes toward the need for a regular PCP, or lack of familiarity or understanding of primary care.^30^

Our results are reasonably precise and can rule out effects larger than a 0.9% point increase in primary care engagement as a result of the intervention with 95% confidence. The intervention took place during the second year of the COVID-19 pandemic, when people may have been wary to seek health care due to fears of contracting COVID-19. However, with respect to internal validity, we would not expect any pandemic-related suppression in health care use to be differential between intervention and control groups. With respect to external validity, some caution should be taken in generalizing these findings to other states given the contemporaneous or unique factors that overlapped with the intervention period in Maine, including Maine's recent expansion, the COVID-19 pandemic, the Medicaid continuous coverage provision, and provider backlogs.

Despite known benefits, little empirical research has studied strategies to improve primary care uptake among Medicaid enrollees. One randomized intervention among newly enrolled Medicaid managed care beneficiaries in California randomized tailored text messages about primary care, which also largely had null effects.^31^ Other randomized studies have shown that small cash incentives can increase primary care visits among low-income adults.^32,33^ Given the complexity of arranging and attending a primary care visit, higher-touch strategies, such as direct financial incentives or outreach from a physician's office that could assist with intake and scheduling, may strengthen the effects of mail or text-based interventions.

Strategies to promote primary care use among the newly insured are often employed by health plans. Some Medicaid MCOs use welcome phone calls at the outset of enrollment to build trust between the member and the MCO, while others rely on partnerships with community-based organizations (CBOs) to identify, engage, and connect members with primary care services.^16,34,35^ However, while these plan-level interventions have face validity, their effectiveness have not been systematically evaluated. Additionally, Maine does not use managed care contracts to deliver services to MaineCare enrollees. As of July 2022, Maine's PCCM program was rolled into PCPlus, a new model in which participating providers receive risk-adjusted population-based payments. In this model, enrollees are automatically assigned a primary care provider based on where they’ve received the plurality of their MaineCare primary care claims.

Previous work has found that utilization of primary care is lower in Medicaid than in Marketplace insurance, likely due to a number of possible factors, including limited access to primary care in Medicaid, lower barriers to emergency department use, and underlying health and demographic characteristics of the 2 populations.^36-39^ Identifying strategies to overcome these challenges will be essential as primary care is central to many growing value-based payment initiatives in Maine and other states.^40^

### Policy implications

Our findings have implications for the provisions in the One Big Beautiful Bill Act of 2025 that included the passage of work and community engagement requirements for those enrolled in the Affordable Care Act's Medicaid expansion. These program changes will require states to communicate frequently with enrollees to prevent confusion and inappropriate coverage losses. Our findings suggest that mail-based strategies may not be effective for behavior change (ie, if enrollees need to act upon the information provided in the letter). Higher-touch strategies, such as partnerships with community-based organizations that can provide more targeted messaging and support, or a dedicated hotline for questions, may prove more effective for preventing unnecessary coverage loss, but would be costlier for state budgets. Our findings suggest that the “opt out” framework in our study reduced administrative burden on enrollees, which could be applied to other procedures at renewal, such as pre-populating information from the prior renewal or other data sources, shortening the application time and reducing administrative burden.

Our results found no significant effects of a mail-based intervention on primary care engagement that assigned a PCP to Medicaid enrollees, though results did suggest the intervention did reduce the administrative burden of contacting MaineCare to designate a PCP. More research is needed to evaluate the relative cost-effectiveness of outreach strategies to communicate with Medicaid enrollees to inform allocation of Medicaid dollars, particularly in light of significant Medicaid policy changes in the OBBBA.

## Supplementary Material

qxag100_Supplementary_Data
