# Supplementary material for: Randomized communication nudges to increase primary care engagement among Medicaid enrollees in Maine
Source: Health Aff Sch. 2026 Jun 24;4(6):qxag100. doi: 10.1093/haschl/qxag100 (PMC13293377; doi:10.1093/haschl/qxag100)
Supplement: qxag100_Supplementary_Data [file qxag100_supplementary_data.zip › Appendix_clean_4_27_26.docx]

Appendix for “Randomized Member Communication Strategies to Increase Primary Care Engagement in Medicaid”

Appendix 1…………………………………Example Mailers

Appendix 2…………………………………MaineCare Randomization Protocol

Appendix 3…………………………………Definition of Outcomes

Appendix 4…………………………………CONSORT Diagram

Appendix 5…………………………………Unadjusted Chi-Square Comparisons of Primary Outcomes Between Intervention and Control Groups in MaineCare Primary Care Case Management (PCCM)

Appendix 6………………………………..Effects of Randomized Communication Nudge by Rurality of MaineCare Enrollee Residence

**Appendix 1. MaineCare PCCM Primary Care Reminders**

*7-Day Mailer*

|  |  |
| --- | --- |
|  |  |


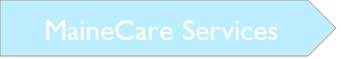


<CH FName LName> August 18, 2020

<Address1>

<Address2>

<City>, <State> <Zip>

Dear <CH FName>,

<FName LName> is enrolled in MaineCare with a program that helps manage your health care needs. Developing a relationship with a Primary Care Provider (PCP) is a step toward better health. You have been assigned to the PCP below.

<Provider Name>

<Practice Name>

<Physical Address>

<Phone>

What do I do next?

If this is the PCP you want to see, call them today about an appointment.

- There should be no cost, unless you have a copay
- Your PCP will get to know you and your medical history
- Bring your MaineCare card to all appointments

If this isn’t your PCP or you would like to choose a different PCP, do one of the following by <Month DD, YYYY>:

- Write the name on the enclosed response card and mail it back to us
- Email mainecaremember@DXC.com
- Call 1-800-977-6740. TTY users dial 711 for Maine Relay

How can my PCP help me?

- Manage all your healthcare needs
- Provide preventive care, such as tests and immunizations, to help keep you well
- Refer you (give you the OK) to see another doctor or specialist for services your PCP doesn’t provide
- Available 7 days a week, 24 hours a day to ask urgent questions about your health needs
  - If the PCP can’t see you right away, you can go to an urgent care center
  - If you have an emergency, you can go to the emergency department

What if I want more information?

- For more information, call MaineCare Member Services at 1-800-977-6740. TTY users dial 711 for Maine Relay. Office hours are Monday - Friday, 7:00 a.m. to 6:00 p.m.
- For information about MaineCare or PCCM, go to the MaineCare Member Handbook at https://www.maine.gov/dhhs/sites/maine.gov.dhhs/files/inline-files/mainecare-member-handbook.pdf.


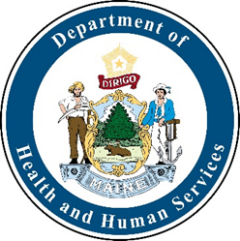

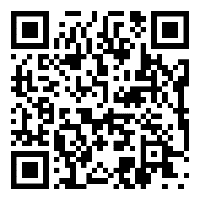


*14 Day Mailer*

|  |  |
| --- | --- |
|  |  |


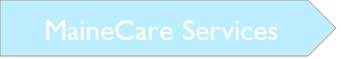


<CH FName LName> August 18, 2020

<Address1>

<Address2>

<City>, <State> <Zip>

Dear <CH FName>,

<FName LName> has health insurance with MaineCare. Connecting with a primary care provider (PCP) is the next step to keeping you healthy!

It’s time to call your PCP about an appointment:

<Provider Name>

<Practice Name>

<Physical Address>

<Phone>

Check with your PCP today. You may be able to see them from the comfort of your own home.

If this isn’t your PCP or you would like to choose a different PCP, do one of the following by <Month DD, YYYY>:

- Write the name on the enclosed response card and mail it back to us
- Email mainecaremember@DXC.com
- Call 1-800-977-6740. TTY users dial 711 for Maine Relay


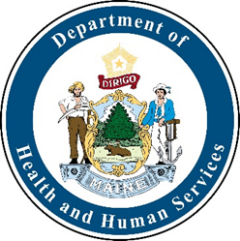


*Enclosed Return Mailer*

***
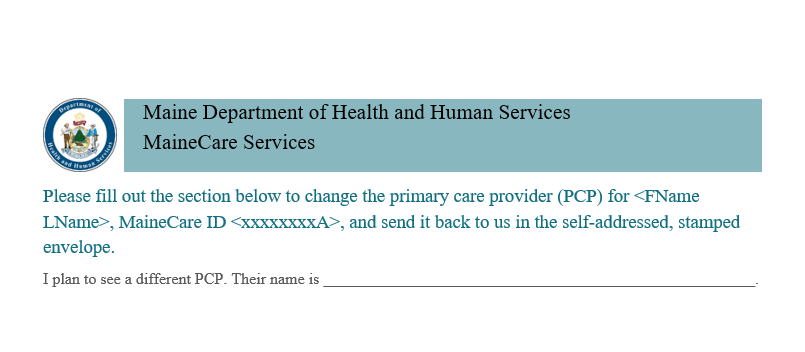
***

**Appendix 2: MaineCare Randomization Protocol**

This intervention was a collaborative effort between researchers at Boston University and the Office of MaineCare Services. The randomization protocol we implemented was as follows, and was effectuated as planned:

- Every Tuesday by 10am during the trial period, MaineCare administrators pulled a report of new PCCM members. Those who were enrolled in PCCM less than one year ago, who received standard enrollment packets more than 1 day before the report, and who were under the age of 19 were removed.
- Member IDs were then anonymized and sent to the academic partners at Boston University for randomization in batch form, corresponding to each week of enrollment.
- The academic partners send the list back to MaineCare by 5pm with treatment or control group status randomly assigned for each new member based on a computerized randomization algorithm using Stata.
- Before day 7 following the enrollment packet mailing, the active Medicaid provider database was used to assign those in the treatment group a PCP who was taking new patients nearest to the enrollee’s home. If multiple providers qualified across participants, assignment was rotated across providers to balance new patient load.
- Information was populated in mailers and sent to enrollees in treatment arm by business day 7.
- Those who had not yet contacted MaineCare were then sent a 14-day reminder card.
- Member responses via phone, email, or mail were tracked by MaineCare administrators for the duration of the intervention period.

**Appendix 3: Definitions of Study Outcomes**

Methodology for defining primary care visits in MaineCare claims

# **Outcome Definitions**

| **Primary Outcome Measures** | **Definition and Relevant Codes** | **Notes** |
| --- | --- | --- |
| Primary care visit | Annual rate of primary care visits occurring within 90, 365 days of enrollment in MaineCare. | We followed the approach to identifying primary care visits outlined by the Maine Quality Forum in their annual report on primary care spending in Maine. This report is required by law and has carefully refined an approach to primary care spending in the state based on the Maine All Payer Claims Database which includes MaineCare claims.  We applied the broad definition of primary care used in this report which includes all billed services provided by identified primary care providers (e.g., family medicine, internal medicine, pediatrics, FQHCs, nurse practitioners) irrespective of specific procedure types as our primary approach. We also tested a narrower definition of primary care use which included only certain primary care services (e.g., specific office visit or preventive procedure codes) performed by primary care providers, but our intervention results did not change. |
| Ambulatory-care sensitive admissions | Annual rate of ambulatory-care sensitive admissions after enrollment in MaineCare. Conditions and relevant diagnosis codes in **Appendix Table 2**. | Based on the Billings NYU classification system and Agency for Healthcare Research and Quality (AHRQ) Prevention Quality Indicators. |
| Emergency department visits | Annual rate of emergency department visits after enrollment in MaineCare. | Measured using Admission Type (1), Revenue Code (0450-0459, 0981), CPT Codes (99281-99285), and Facility Type (23) |
| All-cause inpatient hospitalization | Annual rate of inpatient hospitalizations after enrollment in MaineCare |  |
| **Secondary Outcome Measures** |  |  |
| Any loss of MaineCare coverage per year | At least one month without MaineCare coverage |  |
| Annual flu shots | CPT: 90630, 90653, 90654, 90655, 90656, 90657, 90658, 90659, 90660, 90661, 90662, 90664, 90666, 90667, 90668, 90672, 90673, 90674, 90682, 90685, 90686, 90687, 90688, 90724 | Measured using Current Procedural Terminology (CPT) Codes and HCPCS |
| Annual cholesterol screening | CPT: 80061, 82465, 83718, 84478, 83719  ICD-10: Z13.220, 83721, E78.1, E78.2, 83700, 83701, 83704 | Measured using CPT and ICD-10 codes |
| Mammogram | CPT: 77063, 77067, 77065, 77066, 77062, 77061  ICD-10: G0202, G0204, G0206 | Limited to females 50-64  Measured using CPT and ICD-10 codes |
| Chlamydia testing | 87491, 87591, 87110, 87270, 87320, 87490, 87491, 87810 | Limited to patients ages 19-24;  Measured using CPT codes |
| A1C for enrollees with diabetes | CPT: 83036, 82950, 82947, 83037 | Limited to patients with diagnosis of diabetes (ICD-10 E08.00-E13.9)  Measured using CPT codes |
| Microalbumin for enrollees with diabetes | CPT: 82570, 82043 | Limited to patients with diagnosis of diabetes ICD-10 E08.00-E13.9)  Measured using CPT codes |

**Definition of Ambulatory Care Sensitive Conditions**

| **Condition** | **Comments** | **ICD-10 Codes** |
| --- | --- | --- |
| Congenital syphilis | Secondary diagnosis for newborns only | A50 |
| Immunization-related and preventable conditions | Hemophilus meningitis [G002] age 1-5 only | A33, A34, A35, A37, A80, G000, I01 |
| Grand mal status and other epileptic convulsions |  | G40 |
| Convulsions "A" | Age 0-5 | R56 |
| Convulsions "B" | Age >5 | R56 |
| Severe ENT infections | Exclude otitis media cases [H66, H67] with myringotomy with insertion of tube [Z9622] | H66, J02, J03, J06, J312 |
| Pulmonary tuberculosis |  | A150, A155, A159 |
| Other tuberculosis |  | A154, A156, A158, A17, A18, A19 |
| Chronic obstructive pulmonary disease | Acute bronchitis [J209] only with secondary diagnosis of J41, J42, J43, J44, J47 | J20, J40, J41, J42, J43, J44, J47 |
| Bacterial pneumonia | Exclude case with secondary diagnosis of sickle cell [D57] and patients < 2 months | J13, J14, J153, J154, J157, J159, J16, J18 |
| Asthma |  | J45 |
| Congestive heart failure | Exclude cases with a surgical procedure starting with 02 | I50, I110, J810 |
| Hypertension | Exclude cases with a surgical procedure starting with 02 | I10, I119, I16 |
| Angina | Exclude cases with a surgical procedure starting with 0 or 1 | I20, I240, I248, I249 |
| Cellulitis | Exclude cases with a surgical procedure [starting with 0 or 1], except incision of skin and subcutaneous tissue [0H, 0J, 0W, OX] where it is the only listed surgical procedure | L03, L04, L08, L88, L980 |
| Skin grafts with cellulitis | Exclude admissions from SNF/ICF, AP-DRG 263-264; MS-DRG 573-575 | L02, L03, L89, L97, L98 |
| Diabetes "A" |  | E101, E131, E110, E130, E10641, E11641 |
| Diabetes "B" |  | E106, E116, E108, E118 |
| Diabetes "C" |  | E109, E119 |
| Hypoglycemia |  | E162 |
| Gastroenteritis |  | K529, K5289 |
| Kidney/urinary infection |  | N10, N11, N12 |
| Dehydration - volume depletion | Examine principal and secondary diagnoses separately | E86 |
| Iron deficiency anemia | Age 0 - 5 only, and examine principal and secondary diagnoses separately | D501, D508, D509 |
| Nutritional deficiencies | Examine principal and secondary diagnoses separately | E40, E41, E43, E550, E643 |
| Failure to thrive | Age < 1 only | R6251, R6252, R620, R6250 |
| Pelvic inflammatory disease | Women only denominator - exclude cases with a surgical procedure of hysterectomy [0UT] | N70, N73 |
| Dental Conditions |  | K02, K03, K04, K05, K060, K061, K062, K08, K12, K13, M276, A690, K098 |

**Appendix 4: CONSORT Diagram: Flow of MaineCare Primary Care Case Management Enrollees to Receive Randomized Mail-Based Communication Nudges**

Assessed for eligibility = 4,210

Intervention group = 859

Control group = 3,342

Exclude N=1, missing mailer dates

Exclude N=13

Ineligible for Medicaid

Intervention group = 858

Control group = 3,329

**Appendix 5**: **Unadjusted chi-square comparisons of primary outcomes between intervention and control groups in MaineCare Primary Care Case Management (PCCM)**

|  | Intervention | Control | Unadjusted Chi-sq p-value |
| --- | --- | --- | --- |
| **Any Primary Care Utilization within 365 days of mailer** | 63.2% | 66.2% | 0.092 |
| **Any Emergency Department Utilization within 365 days of mailer** | 32.9% | 33.6% | 0.679 |
| **Any Inpatient Hospitalization within 365 days of mailer** | 10.8% | 11.5% | 0.584 |

**Appendix 6: Effects of Randomized Communication Nudge by Rurality of MaineCare Enrollee Residence**

|  | Any Mainecare Contact | | | Annual MaineCare Enrollment Duration (months), | | | Any Coverage Loss | | |
| --- | --- | --- | --- | --- | --- | --- | --- | --- | --- |
| VARIABLES | Coefficient | 95% CI | p-value | Coefficient | 95% CI | p-value | Coefficient | 95% CI | p-value |
| Group flag (ref=control) | -0.164 | -0.203 - -0.126 | 0.000 | -0.889 | -1.208 - -0.570 | 0.000 | -0.002 | -0.021 - 0.018 | 0.881 |
| Rural falg (ref=not rural) | 0.031 | -0.009 - 0.071 | 0.129 | 0.076 | -0.252 - 0.403 | 0.651 | -0.013 | -0.031 - 0.006 | 0.176 |
| Group flag * Rural flag | -0.024 | -0.093 - 0.046 | 0.508 | -0.021 | -0.593 - 0.551 | 0.943 | 0.016 | -0.021 - 0.054 | 0.399 |
| Gender (ref=male) | 0.074 | 0.044 - 0.104 | 0.000 | -0.061 | -0.250 - 0.128 | 0.528 | -0.006 | -0.019 - 0.008 | 0.406 |
| Age | 0.006 | 0.005 - 0.008 | 0.000 | -0.013 | -0.023 - -0.003 | 0.010 | 0.001 | 0.001 - 0.002 | 0.000 |
| Race (ref=white) |  |  |  |  |  |  |  |  |  |
| Black/AA | -0.083 | -0.172 - 0.007 | 0.069 | 0.371 | -0.012 - 0.755 | 0.058 | -0.013 | -0.040 - 0.014 | 0.330 |
| Asian | 0.101 | -0.061 - 0.264 | 0.222 | 0.613 | -0.328 - 1.554 | 0.201 | -0.035 | -0.073 - 0.003 | 0.075 |
| American Indian/Alaskan Native | -0.069 | -0.228 - 0.090 | 0.394 | 1.097 | 0.303 - 1.890 | 0.007 | -0.042 | -0.052 - -0.032 | 0.000 |
| Other race | 0.014 | -0.141 - 0.168 | 0.862 | -1.233 | -2.368 - -0.099 | 0.033 | 0.038 | -0.045 - 0.121 | 0.367 |
| Unknown Race | 0.062 | 0.013 - 0.111 | 0.013 | -0.181 | -0.583 - 0.221 | 0.377 | 0.011 | -0.014 - 0.036 | 0.370 |
| Ethnicity (ref=non-Hispanic) |  |  |  |  |  |  |  |  |  |
| Hispanic | - | - | - | - | - | - | - | - | - |
| unknown ethnicity | -0.050 | -0.125 - 0.025 | 0.190 | -0.220 | -0.864 - 0.423 | 0.502 | 0.033 | -0.007 - 0.074 | 0.108 |
|  |  |  |  |  |  |  |  |  |  |
| Observations | 4,187 |  |  | 4,187 |  |  | 4,187 |  |  |

|  | Primary Care Visit within 90 days of last mailer sent | | | Primary Care Visit within 365 days of last mailer sent | | | ED visit within 90 days of last mailer sent | | |
| --- | --- | --- | --- | --- | --- | --- | --- | --- | --- |
| VARIABLES | Coefficient | 95% CI | p-value | Coefficient | 95% CI | p-value | Coefficient | 95% CI | p-value |
| Group flag (ref=control) | -0.035 | -0.073 - 0.004 | 0.079 | -0.041 | -0.087 - 0.005 | 0.082 | -0.020 | -0.053 - 0.012 | 0.221 |
| Rural falg (ref=not rural) | -0.048 | -0.087 - -0.009 | 0.017 | -0.041 | -0.083 - 0.001 | 0.057 | 0.000 | -0.025 - 0.026 | 0.985 |
| Group flag * Rural flag | 0.068 | -0.006 - 0.141 | 0.070 | 0.045 | -0.033 - 0.122 | 0.257 | 0.040 | -0.015 - 0.094 | 0.153 |
| Gender (ref=male) | 0.102 | 0.074 - 0.129 | 0.000 | 0.131 | 0.103 - 0.159 | 0.000 | -0.003 | -0.022 - 0.016 | 0.764 |
| Age | 0.003 | 0.002 - 0.005 | 0.000 | 0.002 | 0.001 - 0.003 | 0.000 | -0.000 | -0.001 - 0.001 | 0.537 |
| Race (ref=white) |  |  |  |  |  |  |  |  |  |
| Black/AA | -0.001 | -0.048 - 0.046 | 0.964 | -0.087 | -0.157 - -0.016 | 0.016 | 0.069 | -0.005 - 0.142 | 0.066 |
| Asian | 0.116 | -0.022 - 0.254 | 0.100 | 0.022 | -0.094 - 0.138 | 0.710 | -0.097 | -0.149 - -0.045 | 0.000 |
| American Indian/Alaskan Native | 0.058 | -0.094 - 0.211 | 0.454 | 0.035 | -0.099 - 0.170 | 0.608 | 0.093 | -0.026 - 0.213 | 0.125 |
| Other race | 0.029 | -0.148 - 0.206 | 0.749 | -0.012 | -0.163 - 0.139 | 0.879 | 0.133 | -0.023 - 0.290 | 0.095 |
| Unknown Race | 0.001 | -0.058 - 0.060 | 0.976 | -0.008 | -0.063 - 0.048 | 0.783 | -0.010 | -0.046 - 0.025 | 0.577 |
| Ethnicity (ref=non-Hispanic) |  |  |  |  |  |  |  |  |  |
| Hispanic | - | - | - | - | - | - | - | - | - |
| unknown ethnicity | -0.111 | -0.176 - -0.047 | 0.001 | -0.142 | -0.214 - -0.070 | 0.000 | -0.024 | -0.064 - 0.015 | 0.229 |
|  |  |  |  |  |  |  |  |  |  |
| Observations | 4,187 |  |  | 4,187 |  |  | 4,187 |  |  |

|  | ED visit within 365 days of last mailer sent | | | All-Cause Inpatient Hospitalization within 365 days of last mailer sent | | | Ambulatory care-sensitive admission within 365 days of last mailer sent | | |
| --- | --- | --- | --- | --- | --- | --- | --- | --- | --- |
| VARIABLES | Coefficient | 95% CI | p-value | Coefficient | 95% CI | p-value | Coefficient | 95% CI | p-value |
| Group flag (ref=control) | -0.032 | -0.066 - 0.002 | 0.063 | -0.014 | -0.040 - 0.013 | 0.316 | 0.005 | -0.037 - 0.046 | 0.828 |
| Rural falg (ref=not rural) | 0.001 | -0.037 - 0.039 | 0.948 | -0.005 | -0.027 - 0.018 | 0.687 | 0.017 | -0.024 - 0.058 | 0.419 |
| Group flag * Rural flag | 0.078 | 0.014 - 0.142 | 0.017 | 0.025 | -0.026 - 0.076 | 0.335 | -0.029 | -0.095 - 0.038 | 0.398 |
| Gender (ref=male) | 0.017 | -0.011 - 0.045 | 0.230 | 0.052 | 0.034 - 0.071 | 0.000 | -0.029 | -0.057 - -0.001 | 0.045 |
| Age | -0.001 | -0.002 - 0.000 | 0.175 | -0.001 | -0.001 - 0.000 | 0.107 | 0.003 | 0.002 - 0.005 | 0.000 |
| Race (ref=white) |  |  |  |  |  |  |  |  |  |
| Black/AA | 0.076 | -0.005 - 0.157 | 0.066 | 0.073 | -0.007 - 0.152 | 0.072 | 0.052 | -0.050 - 0.155 | 0.316 |
| Asian | -0.169 | -0.284 - -0.054 | 0.004 | -0.042 | -0.116 - 0.033 | 0.271 | -0.059 | -0.079 - -0.038 | 0.000 |
| American Indian/Alaskan Native | 0.171 | 0.006 - 0.337 | 0.042 | -0.064 | -0.137 - 0.009 | 0.084 | -0.038 | -0.056 - -0.019 | 0.000 |
| Other race | 0.167 | 0.004 - 0.331 | 0.045 | 0.019 | -0.092 - 0.131 | 0.737 | -0.037 | -0.054 - -0.019 | 0.000 |
| Unknown Race | -0.036 | -0.085 - 0.012 | 0.142 | -0.023 | -0.053 - 0.008 | 0.139 | -0.010 | -0.041 - 0.021 | 0.534 |
| Ethnicity (ref=non-Hispanic) |  |  |  |  |  |  |  |  |  |
| Hispanic | - | - | - | - | - | - | - | - | - |
| unknown ethnicity | -0.099 | -0.155 - -0.043 | 0.001 | -0.012 | -0.050 - 0.026 | 0.543 | -0.020 | -0.045 - 0.005 | 0.113 |
|  |  |  |  |  |  |  |  |  |  |
| Observations | 4,187 |  |  | 4,187 |  |  | 4,187 |  |  |

**# of mailers (treatment group only)**

| # of mailers sent | Freq. | Percent | Cum. |
| --- | --- | --- | --- |
|  |  |  |  |
| 0 | 38 | 4.44 | 4.44 |
| 1 | 177 | 20.70 | 25.15 |
| 2 | 640 | 74.85 | 100.00 |
|  |  |  |  |
| Total | 855 | 100.00 |  |
|  |  |  |  |

|  | Any Mainecare Contact | | | Annual MaineCare Enrollment Duration (months), | | | Any Coverage Loss | | |
| --- | --- | --- | --- | --- | --- | --- | --- | --- | --- |
| VARIABLES | Coefficient | 95% CI | p-value | Coefficient | 95% CI | p-value | Coefficient | 95% CI | p-value |
| Group flag (ref=control) | -0.805 | -1.009 - -0.601 | 0.000 | -0.055 | -0.076 - -0.035 | 0.000 | -0.039 | -0.422 - 0.345 | 0.842 |
| Rural falg (ref=not rural) | 0.131 | -0.038 - 0.300 | 0.130 | 0.005 | -0.015 - 0.024 | 0.649 | -0.278 | -0.684 - 0.128 | 0.179 |
| Group flag * Rural flag | -0.085 | -0.452 - 0.281 | 0.649 | -0.001 | -0.037 - 0.035 | 0.956 | 0.351 | -0.390 - 1.093 | 0.353 |
| Gender (ref=male) | 0.333 | 0.197 - 0.470 | 0.000 | -0.004 | -0.015 - 0.008 | 0.526 | -0.120 | -0.383 - 0.143 | 0.371 |
| Age | 0.029 | 0.023 - 0.034 | 0.000 | -0.001 | -0.001 - -0.000 | 0.010 | 0.025 | 0.014 - 0.037 | 0.000 |
| Race (ref=white) |  |  |  |  |  |  |  |  |  |
| Black/AA | -0.386 | -0.843 - 0.072 | 0.099 | 0.022 | -0.000 - 0.045 | 0.054 | -0.315 | -1.074 - 0.445 | 0.416 |
| Asian | 0.433 | -0.264 - 1.131 | 0.223 | 0.037 | -0.018 - 0.092 | 0.192 | -1.072 | -2.972 - 0.828 | 0.269 |
| American Indian/Alaskan Native | -0.314 | -1.104 - 0.475 | 0.435 | 0.064 | 0.020 - 0.109 | 0.005 |  |  |  |
| Other race | 0.073 | -0.625 - 0.771 | 0.838 | -0.078 | -0.152 - -0.004 | 0.040 | 0.681 | -0.447 - 1.810 | 0.237 |
| Unknown Race | 0.281 | 0.067 - 0.495 | 0.010 | -0.011 | -0.036 - 0.014 | 0.378 | 0.229 | -0.237 - 0.695 | 0.335 |
| Ethnicity (ref=non-Hispanic) |  |  |  |  |  |  |  |  |  |
| Hispanic | - | - | - | - | - | - | - | - | - |
| unknown ethnicity | -0.221 | -0.552 - 0.109 | 0.190 | -0.014 | -0.054 - 0.026 | 0.500 | 0.476 | -0.119 - 1.070 | 0.117 |
|  |  |  |  |  |  |  |  |  |  |
| Observations | 4,187 |  |  | 4,187 |  |  | 4,153 |  |  |

|  | Primary Care Visit within 90 days of last mailer sent | | | Primary Care Visit within 365 days of last mailer sent | | | ED visit within 90 days of last mailer sent | | |
| --- | --- | --- | --- | --- | --- | --- | --- | --- | --- |
| VARIABLES | Coefficient | 95% CI | p-value | Coefficient | 95% CI | p-value | Coefficient | 95% CI | p-value |
| Group flag (ref=control) | -0.146 | -0.311 - 0.018 | 0.081 | -0.187 | -0.393 - 0.018 | 0.074 | -0.184 | -0.484 - 0.117 | 0.232 |
| Rural falg (ref=not rural) | -0.201 | -0.367 - -0.035 | 0.017 | -0.190 | -0.382 - 0.001 | 0.051 | 0.002 | -0.213 - 0.218 | 0.983 |
| Group flag * Rural flag | 0.287 | -0.022 - 0.596 | 0.069 | 0.205 | -0.140 - 0.550 | 0.244 | 0.336 | -0.111 - 0.784 | 0.140 |
| Gender (ref=male) | 0.428 | 0.313 - 0.542 | 0.000 | 0.593 | 0.463 - 0.722 | 0.000 | -0.024 | -0.183 - 0.135 | 0.766 |
| Age | 0.014 | 0.009 - 0.019 | 0.000 | 0.011 | 0.005 - 0.016 | 0.000 | -0.002 | -0.009 - 0.005 | 0.540 |
| Race (ref=white) |  |  |  |  |  |  |  |  |  |
| Black/AA | -0.004 | -0.197 - 0.190 | 0.969 | -0.391 | -0.692 - -0.090 | 0.011 | 0.492 | 0.039 - 0.946 | 0.033 |
| Asian | 0.475 | -0.098 - 1.048 | 0.104 | 0.115 | -0.481 - 0.711 | 0.705 | -1.354 | -2.695 - -0.014 | 0.048 |
| American Indian/Alaskan Native | 0.242 | -0.374 - 0.859 | 0.441 | 0.161 | -0.493 - 0.816 | 0.629 | 0.620 | -0.045 - 1.285 | 0.068 |
| Other race | 0.123 | -0.608 - 0.854 | 0.742 | -0.056 | -0.731 - 0.619 | 0.871 | 0.849 | 0.046 - 1.652 | 0.038 |
| Unknown Race | 0.006 | -0.239 - 0.251 | 0.964 | -0.039 | -0.289 - 0.211 | 0.762 | -0.088 | -0.403 - 0.227 | 0.585 |
| Ethnicity (ref=non-Hispanic) |  |  |  |  |  |  |  |  |  |
| Hispanic | - | - | - | - | - | - | - | - | - |
| unknown ethnicity | -0.494 | -0.777 - -0.210 | 0.001 | -0.608 | -0.923 - -0.293 | 0.000 | -0.240 | -0.627 - 0.147 | 0.225 |
|  |  |  |  |  |  |  |  |  |  |
| Observations | 4,187 |  |  | 4,187 |  |  | 4,187 |  |  |

|  | ED visit within 365 days of last mailer sent | | | All-Cause Inpatient Hospitalization within 365 days of last mailer sent | | | Ambulatory care-sensitive admission within 365 days of last mailer sent | | |
| --- | --- | --- | --- | --- | --- | --- | --- | --- | --- |
| VARIABLES | Coefficient | 95% CI | p-value | Coefficient | 95% CI | p-value | Coefficient | 95% CI | p-value |
| Group flag (ref=control) | -0.150 | -0.310 - 0.009 | 0.064 | -0.146 | -0.429 - 0.137 | 0.311 | -0.024 | -0.639 - 0.590 | 0.938 |
| Rural falg (ref=not rural) | 0.005 | -0.167 - 0.177 | 0.955 | -0.050 | -0.280 - 0.179 | 0.666 | -0.127 | -0.626 - 0.372 | 0.618 |
| Group flag * Rural flag | 0.350 | 0.068 - 0.631 | 0.015 | 0.259 | -0.244 - 0.761 | 0.313 | -0.096 | -1.189 - 0.998 | 0.864 |
| Gender (ref=male) | 0.078 | -0.050 - 0.206 | 0.231 | 0.537 | 0.346 - 0.728 | 0.000 | -0.457 | -0.825 - -0.090 | 0.015 |
| Age | -0.004 | -0.010 - 0.002 | 0.175 | -0.006 | -0.014 - 0.001 | 0.106 | 0.055 | 0.043 - 0.066 | 0.000 |
| Race (ref=white) |  |  |  |  |  |  |  |  |  |
| Black/AA | 0.324 | -0.007 - 0.654 | 0.055 | 0.565 | 0.054 - 1.076 | 0.030 | 0.457 | -0.364 - 1.278 | 0.275 |
| Asian | -0.902 | -1.674 - -0.130 | 0.022 | -0.478 | -1.507 - 0.551 | 0.363 |  |  |  |
| American Indian/Alaskan Native | 0.701 | 0.038 - 1.365 | 0.038 | -0.822 | -2.095 - 0.452 | 0.206 |  |  |  |
| Other race | 0.691 | 0.036 - 1.346 | 0.039 | 0.175 | -0.779 - 1.129 | 0.719 |  |  |  |
| Unknown Race | -0.165 | -0.391 - 0.060 | 0.151 | -0.255 | -0.616 - 0.105 | 0.165 | -0.014 | -0.699 - 0.671 | 0.968 |
| Ethnicity (ref=non-Hispanic) |  |  |  |  |  |  |  |  |  |
| Hispanic | - | - | - | - | - | - | - | - | - |
| unknown ethnicity | -0.521 | -0.814 - -0.228 | 0.000 | -0.146 | -0.630 - 0.338 | 0.553 | -0.541 | -1.436 - 0.355 | 0.236 |
|  |  |  |  |  |  |  |  |  |  |
| Observations | 4,187 |  |  | 4,187 |  |  | 4,065 |  |  |
